# Mechanisms Underlying the Antidepressant Response of Acupuncture via PKA/CREB Signaling Pathway

**DOI:** 10.1155/2017/4135164

**Published:** 2017-04-16

**Authors:** Huili Jiang, Xuhui Zhang, Yu Wang, Huimin Zhang, Jing Li, Xinjing Yang, Bingcong Zhao, Chuntao Zhang, Miao Yu, Mingmin Xu, Qiuyun Yu, Xingchen Liang, Xiang Li, Peng Shi, Tuya Bao

**Affiliations:** ^1^School of Acupuncture-Moxibustion and Tuina, Beijing University of Chinese Medicine, Beijing 100029, China; ^2^Department of Traditional Chinese Medicine, Beijing United Family Rehabilitation Hospital, Beijing 100016, China; ^3^School of Acupuncture-Moxibustion and Tuina, Shaanxi University of Chinese Medicine, Xianyang 712046, China

## Abstract

Protein kinase A (PKA)/cAMP response element-binding (CREB) protein signaling pathway, contributing to impaired neurogenesis parallel to depressive-like behaviors, has been identified as the crucial factor involved in the antidepressant response of acupuncture. However, the molecular mechanisms associated with antidepressant response of acupuncture, neurogenesis, and depressive-like behaviors ameliorating remain unexplored. The objective was to identify the mechanisms underlying the antidepressant response of acupuncture through PKA signaling pathway in depression rats by employing the PKA signaling pathway inhibitor H89 in in vivo experiments. Our results indicated that the expression of hippocampal PKA-*α* and p-CREB was significantly downregulated by chronic unpredicted mild stress (CUMS) procedures. Importantly, acupuncture reversed the downregulation of PKA-*α* and p-CREB. The expression of PKA-*α* was upregulated by fluoxetine, but not p-CREB. No significant difference was found between Acu and FLX groups on the expression of PKA-*α* and p-CREB. Interestingly, H89 inhibited the effects of acupuncture or fluoxetine on upregulating the expression of p-CREB, but not PKA-*α*. There was no significant difference in expression of CREB among the groups. Conclusively, our findings further support the hypothesis that acupuncture could ameliorate depressive-like behaviors by regulating PKA/CREB signaling pathway, which might be mainly mediated by regulating the phosphorylation level of CREB.

## 1. Introduction

Depressive disorder is a common mental disorder which has been affecting millions of people worldwide [1]. The main symptoms of depressive disorder are characterized by mood disturbances, anhedonia, cognitive dysfunction, or heightened vulnerability to relapse [2]. It has been investigated that stressful factors are the most prevalently precipitating factors for the development, maintenance, or exacerbation of depressive disorder. There is sufficient evidence that stressful factors are closely associated with impaired neurogenesis and structural plasticity in the hippocampus [3, 4]. Furthermore, additional studies have indicated that morphological changes, impaired neurogenesis, damaged structural plasticity, or even apoptosis in the hippocampus were detected in depressive disorder [5–9].

Presently, antidepressant drugs (e.g., paroxetine, fluoxetine) are the major treatment for depression and have been widely used for the treatment of depression in the clinic. Fluoxetine, one of the antidepressant drugs characterized by a selective serotonin (5-hydroxytryptamine (5-HT)) reuptake inhibitor (SSRI), has been approved by the Food and Drug Administration (FDA) to treat stress-related disorders (including depression and anxiety) in patients [10]. Antidepressant drugs are indeed available nowadays. However, it has been evidenced that approximately one third of all patients with depressive disorder fail to respond to conventional antidepressant therapies [11]. Meanwhile, data from clinical investigations and laboratory animals have provided compelling evidence that some antidepressant drugs have anxiogenic effects during the acute phase of treatment or even aggravate suicidal thinking and behavior [10, 12, 13]. Accordingly, there is an urgent need for investigating new conceptual frameworks for understanding the pathogenesis of depression and exploring better treatments for depression.

The precise contributing factors and mechanisms of depression are still unknown. The precise pathogenesis and etiology of depression has been a challenging issue recently. Nowadays, the involvement of alterations concerning immune response and inflammatory response in the pathophysiology of depression and stress has been evidenced by various studies [14–16]. Numerous studies have reported that cAMP-dependent protein kinase- (PKA-) CREB signaling is involved in the pathogenesis of depression. cAMP response element-binding (CREB) protein has been evidenced to be one of the best-studied transcription factors implicated in depression and antidepressant-like process. Data from humans and laboratory experiments have provided compelling evidence that the PKA/CREB signal pathway is involved in the effect on regulating synaptic plasticity and learning memory [17–22]. The systemic perturbations of the PKA/CREB signal pathway could induce cascade reactions of neuropathology in depression, including abnormalities in regional brain activity, alterations in synaptic function, and impaired neurogenesis. Downregulated expression of CREB level has been investigated in the postmortem hippocampus of patients who suffered from depression [23]. CREB signaling has been considered to be a crucial factor implicated in promoting synaptic and neural plasticity by regulating the genes that increase synaptic and neural plasticity, including BDNF [24]. PKA, the upstream activator of CREB, has been evidenced to exhibit antidepressive effect by upregulating CREB or p-CREB [25, 26].

During our previous studies, we have been focusing on investigating the clinical effects and mechanisms of acupuncture on depression. We found distinct abnormalities in regional brain activity [27, 28]. Moreover, data from laboratory animals supporting the involvement of PKA/CREB in the pathogenesis of depression are compelling and include findings that verify the antidepressant response of acupuncture by regulating PKA/CREB [29, 30]. However, the mechanisms underlying the antidepressant response of acupuncture via PKA/CREB have not been investigated in depth.

Here, we established a rat model of depression induced by CUMS and assessed the difference in antidepressant effect between acupuncture and fluoxetine. Importantly, H89, a moderately specific inhibitor for PKA [31, 32], was employed in the present study to investigate the role of CREB or p-CREB, activated by PKA. The expressions of PKA-*α*, CREB, and p-CREB were assessed. We aimed to elucidate the molecular mechanisms underlying the antidepressant response of acupuncture and shed new light on conceptual frameworks of prospects for new therapies in the treatment of depression.

## 2. Materials and Methods

### 2.1. Experimental Animals and Grouping

Adult 6-week-old male Sprague-Dawley (SD) rats, weighing 220 ± 20 g, were obtained from Weitong Lihua Experimental Animal Center of Beijing, China. Rats were housed in a quiet room with a controlled environment of 23°C–26°C and 50% ± 10% humidity. The rats subjected to CUMS were housed separately in different cages for social isolation, and 5 animals per cage were housed for rats in the control group. All experimental procedures were in full observance of the Bioethical Committee of the Institute of Animal Care Committee, Beijing University of Chinese Medicine, Beijing, China (permit no. Kj-dw-32-20150612).

**Figure 1:**
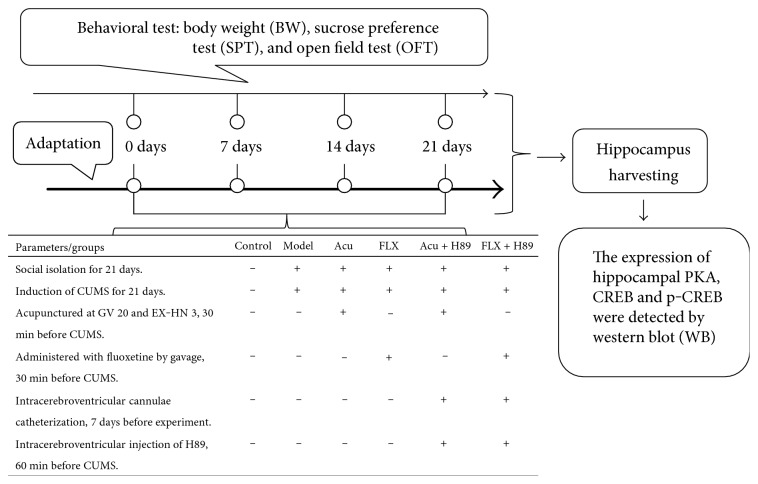
Experimental procedures. Acu: acupuncture group; FLX: fluoxetine group; Acu + H89: Acupuncture + H89 group; FLX + H89: Fluoxetine + H89 group; PKA-*α*: protein kinase A-*α*; CREB: cyclic adenosine monophosphate response element-binding protein; p-CREB: Phosphor-CREB.

The body weight (BW), sucrose preference test (SPT), and open-field test (OFT) were investigated to guarantee the consistency of baseline characteristics before the experimental procedure was conducted. Five rats were excluded due to the inconsistent baseline characteristics. Then, a total of 60 rats under the circumstance of similar baseline characteristics of BW, SPT, and OFT were assigned into control, model, model + acupuncture (Acu), model + fluoxetine (FLX), model + acupuncture + H89 (Acu + H89), and model + fluoxetine + H89 (FLX + H89) groups at random, with 10 rats in each group. All rats were exposed to social isolation and CUMS for 21 days excluding rats in the control group. Thirty minutes before CUMS procedure, the rats in the Acu group were acupunctured at Baihui (GV 20) and Yintang (EX-HN 3); the rats in the FLX group were administered with fluoxetine (0.18 mg/ml) by gavage (1 ml/100 g). Intracerebroventricular injections of the PKA signaling pathway inhibitor H89 (10 *μ*M, 5 *μ*l) were administered in Acu + H89 and FLX + H89 groups 60 minutes before the CUMS procedure, once every other day, and then, acupuncture stimulation and intragastric administration of fluoxetine were conducted, respectively (Figure 1).

### 2.2. Chronic Unpredictable Mild Stress (CUMS)

A depressive disorder model induced by CUMS in rats was established in this study as described previously [29, 33]. Furthermore, some adjustments were made to add the unpredictability. The rats were exposed to CUMS for 21 days, including restricted access to food deprivation for 24 h, water deprivation for 24 h, housing in a wet cage for 24 h (containing 100 g of sawdust in 200 ml water), continuous overnight illumination for 12 h, restricted access to chronic restraint stress for 2 h (restraining in a cylinder-shaped wire net, 20 cm in length and 5 cm in diameter), shaking once per second for 30 min, and clip tail for 3 min (1 cm apart from the tail). Rats were subjected to one of these 7 stimuli at random per day, and the same stressor was not employed on consecutive days to avoid the rat's prediction. Each stressor was used 3 times randomly (Table 1). Rats in the control group were normally fed for 21 days with food and water ad libitum without any stimulus.

### 2.3. Surgical Procedures for Intracerebroventricular (ICV) Catheterization

Rats in the Acu + H89 and FLX + H89 groups were subjected to surgical procedures for intracerebroventricular (ICV) cannulae implanted 7 days before the experimental design as described by previous studies [34, 35]. Rats were anesthetized with 10% chloral hydrate (0.35 ml/100 g, i.p.) and then placed on a stereotaxic apparatus (catalogue no. 68001, RuiWoDe Life Science Co., Ltd., Shenzhen, China). The fixed positions were (coordinates: −0.8 mm from the bregma, ±1.5 mm lateral from sagittal suture, and −1.9 mm deep from the dura) referred to the atlas of the previous study [36, 37]. ICV cannulae (OD 0.56 × ID 0.38 mm/M 3.5, RuiWoDe Life Science Co., Ltd., Shenzhen, China) were inserted bilaterally into the ventricle. Upon screening and confirming outflow of cerebrospinal fluid from the ICV cannulae, the guide cannula was secured with screws and dental cement and closed with a dummy cannula. Then, all rats were sent to a relatively warm room for better recovery for 7 days from surgery, so as to become accustomed to the procedures of further experimentation.

### 2.4. Intracerebroventricular Injection of PKA Inhibitor H89

In the microinfusion experiments, the ICV cannulae were strictly disinfected with 75% alcohol. Microinfusion probes were gently inserted through the guide cannulae. The solution of PKA inhibitor H89 (dissolved with 0.9% sodium chloride solution, 10 *μ*M), 5 *μ*l, was infused bilaterally into the encephalocele at a rate of 0.25 *μ*l/min for 2 min, once every two days. Then, the infusion probes were left in place for an additional 2 min to allow solutions to diffuse away from the probe tips. After redisinfection with 75% alcohol, the ICV cannulae were closed with a dummy cannula.

### 2.5. Acupuncture Stimulation

For the animals that received acupuncture stimulation, acupuncture commenced 30 minutes before CUMS procedure, 10 minutes per session, and 1 session daily for 21 days. After disinfection of the acupoint sites with 75% alcohol, the acupuncture needles (0.3 mm in diameter and 25 mm long; Suzhou Acupuncture & Moxibustion Appliance Co., Ltd., Jiangsu, China) were inserted transversely (keeping the angle between the needle and the skin surface at 15°) into Baihui (GV 20) and Yintang (EX-HN 3) (acupoint coordinates [38]: GV 20, located at the bregma or on the junction of coronal suture and sagittal suture; EX-HN 3: midway between the medial ends of the two eyebrows) to a depth of 5 mm as described by our previous study [29]. When acupuncture procedure was conducted, rats were placed in separated room and under the conditions of free activities.

### 2.6. ICV Catheterization Assessment

To verify the effectiveness and validity of ICV injection of H89 in rats in the Acu + H89 and FLX + H89 groups, another 6 rats were subjected to ICV catheterization and ICV injection of 1% Evans blue. Briefly, rats that received posttraining infusions of 0.9% sodium chloride solution after successful ICV catheterization were returned to the holding cage for better recovery for 7 days from surgery. Then, 0.1% Evans blue (dissolved with deionized water, 5 *μ*l) was injected into the lateral ventricle. After being exposed to free activity for 4 h, rats were deeply anesthetized with 10% chloral hydrate (0.35 ml/100 g, i.p.) and then perfused intracardially with 100 ml of 0.9% sodium chloride solution followed by 200 ml of 4% paraformaldehyde in 0.1 M phosphate-buffered saline (PBS). Then, rats were decapitated, and the brains were removed and placed into 4% paraformaldehyde in 0.1 M PBS for 72 h at 4°C. The brains were transferred to 20% and 30% sucrose solutions for dehydration at 4°C. The morphology of lateral ventricle was detected and visualized under a laser confocal scanning microscope (FV1000, Olympus, Japan) through frozen sections (8 *μ*m) (CM-1950, Leica, German).

### 2.7. Observation of Rat Behavior

All behavioral tests were conducted under relatively quiet and dark circumstances. Body weight (BW), sucrose preference test (SPT), and open-field test (OFT) were investigated at least 12 hours after the stress stimulation at the end of experimental period. Mood states, quality of feces, and appetite of rats were observed.

#### 2.7.1. Body Weight (BW)

The changes in body weight gain in comparison to the baseline were calculated to evaluate the states of food preference and nutrition status. Body weight was detected on day 0 and day 21 for each rat throughout the experimental procedures.

#### 2.7.2. Open-Field Test (OFT)

Locomotor activity of each rat was detected through open-field test (OFT) as illuminated by previous studies [29, 39]. The open-field apparatus consisted of a 80 cm × 80 cm × 40 cm square arena with black wall and black base, of which the base was divided into 16 × 16 cm equal squares with legible white lines. Each rat was gently placed in the center of the open-field floor and then allowed to enjoy independent movement and explore freely for 5 minutes. Crawling square numbers (numbers of crossing the horizontal sectors including three paws in the same square) and standing times (numbers of erection including rearing) were monitored and recorded as an index of locomotion activity and exploratory behavior. After each trial, 75% ethyl alcohol was used to refresh the open-field apparatus, which could get rid of the interference of odor signals. OFT was conducted on day 0 and day 21.

#### 2.7.3. Sucrose Preference Test (SPT)

Referring to investigations of recent studies [29, 33], sucrose preference test (SPT) was employed to evaluate the condition of anhedonic-like behaviors of rats. Rats were trained to adapt to 1% sucrose solution (Amresco, USA) during the adaptation cycles. After the adaptation, all rats were deprived of food and water for 23 hours. Then, they were all housed in individual cages and had free access to two preweighed bottles containing 150 ml sucrose solution (1% w/v) and 150 ml pure water for 1 hour. At the end of the test, the bottles of 1% sucrose solution and pure water were reweighted and recorded. SPT was conducted on day 0 and day 21. Anhedonia was expressed by reduced sucrose consumption.

### 2.8. Western Blot

Following the last experimental procedure, the rats were sacrificed and decapitated. The brains were removed quickly, and the hippocampus was isolated and stored at −80°C for the next process. The samples were homogenized with RIPA lysis buffer, containing 50 mM Tris (pH 7.4), 150 mM NaCl, 1% NP-40, and 0.5% Na deoxycholate and protease inhibitor cocktail (or phosphatase inhibitor cocktail for phosphorylated protein) for protein extraction. And then, the supernatant was collected following centrifugation at 13,000 rpm at 4°C for 20 minutes. The total protein content was determined by using bicinchoninic acid (BCA) assay. Following the quantitative determination of the total protein content, the proteins of each sample were denatured at 100°C for 5 min and fractionated through 10% SDS polyacrylamide gel electrophoresis (PAGE). The proteins of samples were electrotransferred onto polyvinylidene difluoride membranes with voltage at 80 V for 60 min. The membranes were blocked with 5% bull serum albumin- (BSA-) TBST for 1 h at room temperature. Protein expression was subsequently detected by incubation with rabbit polyclonal primary antibodies against PKA-*α* (1 : 2000; 5842s, Cell Signaling Technology, USA), p-CREB (1 : 500; ser133-9198S, Cell Signaling Technology, USA), CREB (1 : 500; ser133-4820S, Cell Signaling Technology, USA), and GAPDH (1 : 1000; 2118s, Cell Signaling Technology, USA) at 4°C overnight. Following incubation with the primary antibody, the membranes were incubated with goat anti-rabbit HRP-conjugated IgG (1 : 2000; ZDR-5118, Zhongshan Jinqiao Biotechnology Co., Ltd, Beijing, China) at room temperature for 60 min. The bound antibodies were visualized using an enhanced chemiluminescence reagent by ECL kit (RPN2232; GE Healthcare Life Sciences, UK) and quantified densitometrically using Gel-image analyzing system (Gene Gnome, Syngene, USA). The experiments were performed in triplicates with triplicate samples. The mean optical density value of each protein band relative to that of the glyceraldehyde-3-phosphate dehydrogenase (GAPDH) band from the same sample was calculated.

### 2.9. Statistical Analysis

All data were statistically analyzed by SPSS 22.0 software (IBM, Armonk, NY, USA) and expressed as the mean ± standard deviation ($\bar{x}\pm s$). The total sucrose consumption, body weight gain, and expression of PKA-*α*, CREB, and p-CRE were analyzed by a one-way analysis of variance (ANOVA) test. Differences between individual means were tested for significance using Fisher's least significant difference (LSD) or Tamhane's T2 procedure. The horizontal and vertical motion scores were analyzed by the Kruskal-Wallis H test. Probability values less than 0.05 were considered greatly significant.

## 3. Results

### 3.1. Acupuncture Alleviates Depressive-Like Behaviors in Depression Rats Induced by CUMS

**Figure 2:**
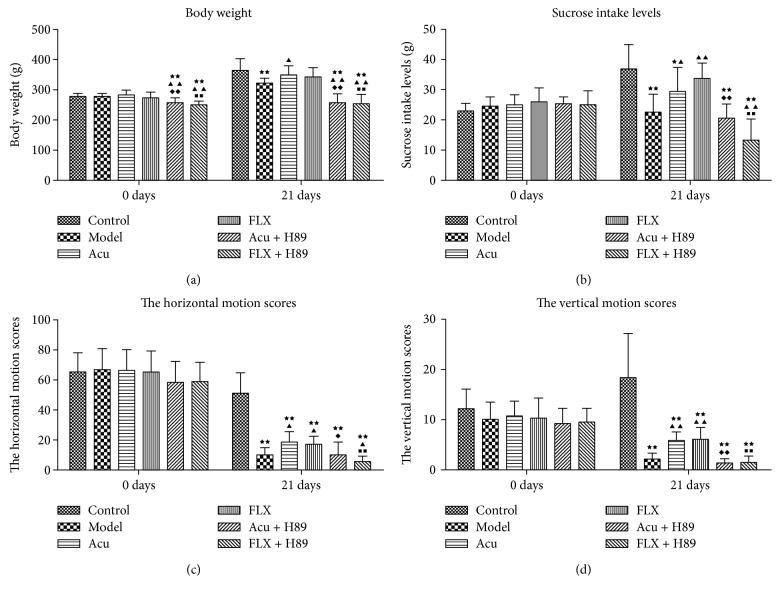
Differences showing the effects of stress/ antidepressant treatments on depressive-like behaviors in depression rats induced by CUMS. (a) The effects of stress/antidepressant treatments on the body weight in depression rats induced by CUMS. (b) The effects of stress/antidepressant treatments on the sucrose intake levels in depression rats induced by CUMS. (c) The effects of stress/antidepressant treatments on the horizontal motion scores in depression rats induced by CUMS. (d) The effects of stress/antidepressant treatments on the vertical motion scores in depression rats induced by CUMS. Differences are shown as follows: ^★^*P* < 0.05 versus control group; ^★★^*P* < 0.01 versus control group; ^▲^*P* < 0.05 versus model group; ^▲▲^*P* < 0.05 versus model group; ^◆^*P* < 0.05 versus acupuncture group; ^◆◆^*P* < 0.01 versus acupuncture group; ^■■^*P* < 0.01 versus fluoxetine group. Values are given as $\bar{x}\pm s$ for 10 rats in each group. Acu: acupuncture group; FLX: fuoxetine group; Acu + H89: Acupuncture + H89 group; FLX + H89: Fluoxetine + H89 group.

To investigate the effects of acupuncture on the depressive-like behaviors in the rat model of depression induced by CUMS, the BW, SPT, and OFT of pre-experiment versus postexperiment were observed. The results showed that, compared with the control group, the gain in body weight was significantly prolonged and less, and the sucrose intake and the times of horizontal and vertical motion scores were notably reduced in the model group, all with statistical significance (*P* < 0.01, *P* < 0.01, *P* < 0.01, and *P* < 0.01) (Tables 2(a), 2(b), and 2(c); Figures 2(a), 2(b), 2(c), and 2(d)). However, the gain in body weight was evidently increased, and the sucrose intake and the times of horizontal and vertical motion scores were notably elevated when the Acu and FLX groups versus those of the model group following treatment with acupuncture and fluoxetine (Acu versus model with statistical significance: *P* < 0.05, *P* < 0.05, *P* < 0.05, and *P* < 0.01) (FLX versus model with statistical significance: *P* < 0.05, *P* < 0.01, *P* < 0.05, and *P* < 0.01) (Tables 2(a), 2(b), and 2(c); Figures 2(a), 2(b), 2(c), and 2(d)). Both acupuncture and fluoxetine could well alleviate the depressive-like behaviors induced by CUMS.

Furthermore, to identify the effects underlying the antidepressant response of acupuncture through PKA pathway in rats exposed to CUMS by employing the PKA signaling pathway, the inhibitor H89 was performed as intracerebroventricular injection to specifically inhibit the PKA signaling pathway. As the results indicated, compared with the control group, the gain in body weight was significantly reduced in the Acu + H89 and FLX + H89 groups' pre-experiment (*P* < 0.01; *P* < 0.01), which might be due to the anesthetization during ICV catheterization surgery disturbing the regular diet order. After 7 days of recovery from surgery, the baseline characteristic of behaviors of rats in the Acu + H89 and FLX + H89 groups kept pace with the others. Then, the BW, SPT, and OFT of pre-experiment versus postexperiment were observed. The results showed that, compared with the Acu group, the gain in body weight and the sucrose intake were significantly reduced (*P* < 0.01; *P* < 0.01), and the times of horizontal and vertical motion scores were notably reduced in the Acu + H89 group, all with statistical significance (*P* < 0.05 and *P* < 0.01) (Tables 2(a), 2(b), and 2(c); Figures 2(a), 2(b), 2(c), and 2(d)). Interestingly, the difference between FLX and FLX + H89 was inconsistent with the results of Acu versus Acu + H89. The antidepressive effects of acupuncture and fluoxetine were both inhibited by PKA inhibitor H89.

### 3.2. ICV Catheterization Assessment

**Figure 3:**
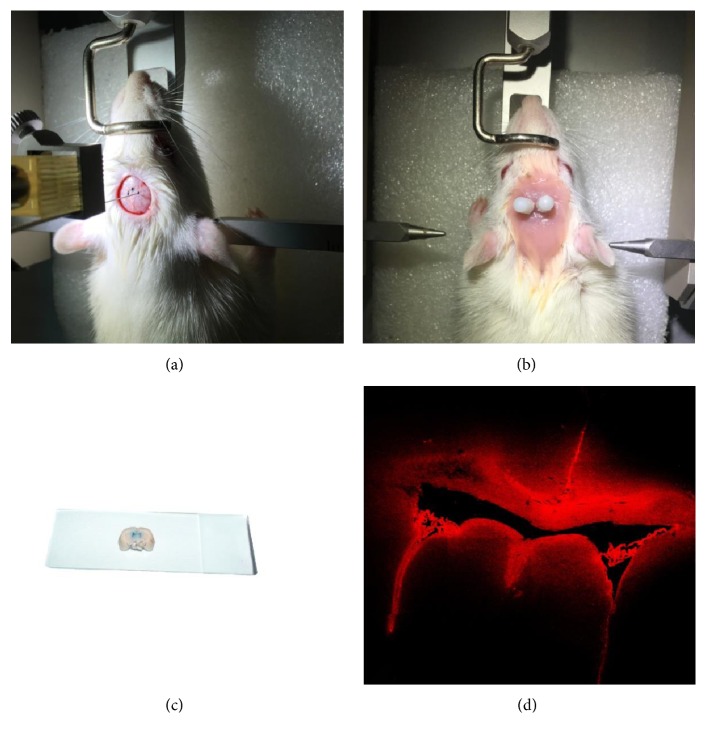
The effectiveness and validity of ICV catheterization. (a) The fixed position of ICV catheterization. (b) The rat subjected to effective ICV catheterization. (c) The structure and morphology of bilateral paracele detected with the naked eye. The bilateral paracele was suffused with blue substance after ICV injection of 1% Evans blue, suggesting the validity of ICV injection and ICV catheterization. (d) The structure and morphology of bilateral paracele screened under the fluorescence microscope. The wall of the whole bilateral paracele, including frontal, occipital, temporal and inferior horn, was suffused with Evans blue exhibiting red fluorescence.

Rats were subjected to ICV catheterization (Figures 3(a) and 3(b)) as described by previous studies [35, 37]. ICV injection of 1% Evans blue into the bilateral paracele was performed to verify and guarantee the effectiveness and validity of ICV injection of H89 in rats in the Acu + H89 and FLX + H89 groups. The results showed that the bilateral paracele were suffused with blue substance after ICV injection of 1% Evans blue when detected with the naked eye (Figure 3(c)). Meanwhile, the structure and morphology of bilateral paracele were investigated by frozen sections and screened under the fluorescence microscope. We found that the wall of the whole bilateral paracele, including frontal, occipital, temporal, and inferior horn, was suffused with Evans blue exhibiting red fluorescence (Figure 3(d)). Accordingly, the effectiveness and validity of ICV catheterization and ICV injection have been notably evidenced by our present study.

### 3.3. The Analysis of Antidepressive Effects of Acupuncture on the PKA/CREB Signaling Pathway

PKA signaling pathway has been evidenced to be associated with the pathogenesis of mental disorders, including depression. In the present study, the expression levels of PKA-*α*, CREB, and p-CREB were detected by western blot analysis to investigate the mechanisms through which acupuncture ameliorates depressive-like behaviors in rats exposed to CUMS. H89, the inhibitor of PKA, was used to explore whether PKA signaling pathway was involved in the antidepressive effects of acupuncture, which in turn investigate the mechanisms underlying the antidepressant response of acupuncture via PKA signaling pathway.

#### 3.3.1. Expression Level of PKA-α in the Hippocampus

**Figure 4:**
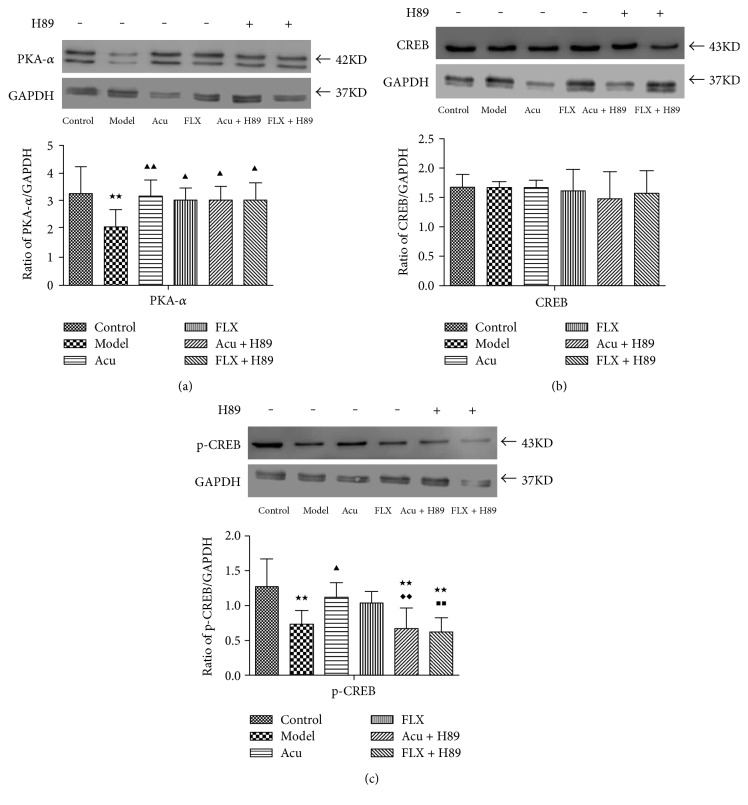
Differences showing the effects of stress/antidepressant treatments on the expression levels of hippocampal PKA-*α*, CREB, and p-CREB in depression rats induced by CUMS. (a) The effects of stress/antidepressant treatments on the expression level of hippocampal PKA-*α* in depression rats induced by CUMS. (b) The effects of stress/antidepressant treatments on the expression level of hippocampal CREB in depression rats induced by CUMS. (c) The effects of stress/antidepressant treatments on the expression level of hippocampal p-CREB in depression rats induced by CUMS. Differences are shown as follows: ^★★^*P* < 0.01 versus control group; ^▲^*P* < 0.05 versus model group; ^▲▲^*P* < 0.01 versus model group; ^◆◆^*P* < 0.01 versus acupuncture group; ^■■^*P* < 0.01 versus fluoxetine group. Results are presented as $\bar{x}\pm s$ for 10 rats in each group. Acu: acupuncture group; FLX: fluoxetine group; Acu + H89: Acupuncture + H89 group; FLX + H89: Fluoxetine + H89 group; PKA-*α*: protein kinase A-*α*; CREB: cyclic adenosine monophosphate response element-binding protein; p-CREB: Phosphor-CREB; GAPDH: glyceraldehyde-3-phosphate dehydrogenase.

The results showed that, compared with control group, the expression of hippocampal PKA-*α* in the model group was significantly downregulated (*P* < 0.01) (Table 3 and Figure 4). Following the treatment of acupuncture and fluoxetine, the expression of hippocampal PKA-*α* in the acupuncture group and fluoxetine group was significantly upregulated compared with that in the model group (*P* < 0.01; *P* < 0.05) (Table 3 and Figure 4). Both acupuncture and fluoxetine could reverse the downregulation of hippocampal PKA-*α* induced by CUMS. No significant difference was found between acupuncture and fluoxetine groups (*P* > 0.05). Similarly, no significant differences were found between the Acu group versus Acu + H89 group and the FLX group versus FLX + H89 group (*P* > 0.05; *P* > 0.05).

#### 3.3.2. Expression Level of CREB and p-CREB in the Hippocampus

Furthermore, expressions of hippocampal CREB and p-CREB were assessed. The results showed that there were no significant differences among the control group, model group, Acu group, FLX group, Acu + H89 group, and FLX + H89 group. However, the p-CREB expression level in the model group was notably decreased compared with that in the control group (*P* < 0.01) (Table 3 and Figure 4). Similarly, the expression of p-CREB was notably downregulated between Acu + H89 versus control group and FLX + H89 versus control group (*P* < 0.01; *P* < 0.01) (Table 3 and Figure 4). By contrast, the p-CREB expression level in the acupuncture group was significantly upregulated compared with that in the model group (*P* < 0.01). While the expression of p-CREB in the fluoxetine group presented a trend of escalation in comparison with the model group (*P* > 0.05). No significant difference was found between the acupuncture and fluoxetine groups. The expression of p-CREB was notably downregulated between the Acu group versus Acu + H89 group and the FLX group versus FLX + H89 group (Table 3 and Figure 4).

## 4. Discussion

The present study aimed to identify molecular and neurobiological mechanisms responsible for antidepressant response following the treatment of acupuncture. A rat model of depressive disorder induced by CUMS was established. Open-field test (OFT), sucrose consumption, and body weight were employed to evaluate the depression-relevant behaviors, including the ability to adapt to new environments, the sensitivity to reward stimulation and pleasure, or mainly the state of anhedonia. Our findings indicated that the rats subjected to CUMS were observed to exhibit obviously poor appetite and significantly slow increase in body weight, the adaptive regression to new environments, the stagnancy to reward stimulation and pleasure, or altered mood switching from irascibility to low spirits throughout the CUMS procedure. The result of the present study is consistent with the current study illustrated that CUMS could induce depressive-like behaviors, effectively imitating the symptoms of depression in the patients [40, 41].

Acupuncture showed compelling antidepressant effects on ameliorating depression-related behaviors. Importantly, PKA, the CREB upstream regulator, and the phosphorylation of CREB on Ser133 by PKA, p-CREB, also showed a strain-dependent expression pattern. Although PKA and p-CREB expression levels were upregulated following the treatment of acupuncture and fluoxetine, inhibition of PKA-CREB signaling by H89 reversed the upregulation of p-CREB expression level (but not PKA and CREB) and the antidepressant effects on ameliorating depression-related behaviors of acupuncture and fluoxetine, suggesting that both acupuncture and fluoxetine could achieve the antidepressant effects by promoting the phosphorylation of CREB on Ser133 by PKA-CREB signaling. Other studies concerning the CREB signaling have shed light on the potentially promoting effects on neurogenesis implicated in cognitive behaviors or synaptic plasticity function involved in antidepression [42–44]. In the present study, we found that the downregulated expression levels of hippocampal PKA-*α* and p-CREB, induced by CUMS, were reversed by acupuncture. What is more, the upregulated expression level of p-CREB, but not PKA and CREB, was inhibited by H89, indicating that the increased p-CREB expression (phosphorylation of CREB) might be partly attributable to the increased activation of PKA. Although no significant difference was found in acupuncture versus fluoxetine, concerning inhibiting the phosphorylation of CREB (expression of p-CREB) by the PKA inhibitor H89, it is notable that the antidepressive effects of acupuncture on alleviating sucrose intake level and the horizontal motion scores were more compelling than fluoxetine following the administration of H89. All these results might indicate that the antidepressive response of acupuncture is not just dependent on PKA/CREB signaling, which is identical with our previous studies [45].

Acupuncture, one of the conventional therapies in traditional Chinese medicine (TCM), contributes to therapeutic effects by regulating the nervous, endocrine, and immune systems [45] and plays an important role in maintaining normal physiologic state of the organism. Based on the basic theory of TCM, acupoint compatibility plays an important role in the acupuncture prescription, which in turn is directly involved in the therapeutic effect clinically. During our previous studies, the underlying effects and mechanisms of acupuncture on depression have been investigated [46, 47]. Baihui (GV 20) and Yintang (GV 29) are considered to be the optimized acupoint modules in the treatment of depression [46, 47]. According to the basic theory of TCM, Baihui (GV 20) and Yintang (GV 29) are acupoints pertaining to the governor meridian, which has a direct contact with the brain through channels and collaterals [48, 49]. Accordingly, acupuncture at these acupoint modules can dredge channels and regulate the flow of Qi and the blood of the governor meridian, which in turn regulates mentality and alleviates depression [48–50]. Data from clinical investigations and laboratory animals have provided evidence that acupuncture exhibited antidepressant-like efficacy on depression [47, 51, 52]. Our previous studies have investigated that acupunctured at Baihui (GV 20) and Yintang (EX-HN 3) could well alleviate depression by increasing the expression of excitatory neurotransmitter in the hippocampus, attenuating impaired neurogenesis and inhibiting the apoptosis of hippocampal neurons [46, 47].

The present study has been evidenced that the activation of phosphorylation of CREB through the strain-dependent PKA/CREB signaling exhibits compelling antidepressant responses to acupuncture. Further studies will focus on the expression of BDNF and the downstream effector of PKA/CREB signaling and other signaling commonly responsible for individual differences in antidepressant responses between antidepressants and acupuncture, which in turn will elucidate the underlying mechanisms concerning the antidepressant response of acupuncture and explore new prospects of integrated medicine in the treatment of depression.

## Conflicts of Interest

The authors declare that they have no competing interests.

## Figures and Tables

**Table 1:** The CUMS procedure of applied stressors during 1 week.

Days	Duration	Stressor
Day 1	30 minutes	Shaking once per second
Day 2	2 hours	Chronic restraint stress
Day 3	3 minutes	Clip tail
Day 4	24 hours	Housing in a wet cage
Day 5	12 hours	Continuous overnight illumination
Day 6	24 hours	Water deprivation
Day 7	24 hours	Food deprivation

**Table:** (a) Differences in body weight following stress/antidepressant treatments

Group	*n*	0 days	21 days
Control	10	277.01 ± 11.10	363.83 ± 38.06
Model	10	275.24 ± 12.75	319.61 ± 17.95^★★^
Acu	10	281.42 ± 17.26	348.04 ± 31.05^▲^
FLX	10	271.93 ± 20.34	350.99 ± 30.49^▲^
Acu + H89	10	256.58 ± 15.01^★★,▲▲,◆◆^	257.86 ± 28.48^★★,▲▲,◆◆^
FLX + H89	10	250.37 ± 11.57^★★,▲▲,■■^	252.73 ± 31.61^★★,▲▲,■■^

**Table:** (b) Differences in sucrose intake levels following stress/antidepressant treatments

Group	*n*	0 days	21 days
Control	10	22.96 ± 2.52	36.71 ± 8.04
Model	10	24.57 ± 2.98	22.52 ± 5.92^★★^
Acu	10	24.80 ± 3.50	29.29 ± 7.99^★,▲^
FLX	10	25.97 ± 4.64	33.51 ± 5.17^▲▲^
Acu + H89	10	25.19 ± 2.59	20.52 ± 4.57^★★,◆◆^
FLX + H89	10	25.12 ± 1.98	13.39 ± 6.92^★★,▲▲,■■,^^∗^

**Table:** (c) Differences in horizontal and vertical motion scores following stress/antidepressant treatments

Group	0 days		21 days	
	Horizontal	Vertical	Horizontal	Vertical
Control	65.10 ± 13.01	12.10 ± 4.04	50.70 ± 13.84	18.40 ± 8.76
Model	66.60 ± 14.04	10.00 ± 3.53	9.50 ± 4.97^★★^	2.10 ± 1.29^★★^
Acu	65.80 ± 14.40	10.80 ± 2.90	18.10 ± 7.37^★★,▲^	5.80 ± 1.81^★★,▲▲^
FLX	65.20 ± 13.74	10.30 ± 4.01	16.90 ± 5.30^★★,▲^	6.10 ± 2.23^★★,▲▲^
Acu + H89	58.20 ± 13.66	9.20 ± 3.12	10.20 ± 8.20^★★,◆^	1.20 ± 1.03^★★,◆◆^
FLX + H89	58.70 ± 12.69	9.50 ± 2.76	5.30 ± 3.80^★★,▲,■■^	1.50 ± 1.27^★★,■■^

Acu: acupuncture group; FLX: fluoxetine group; Acu + H89: Acupuncture + H89 group; FLX + H89: Fluoxetine + H89 group. Results are presented as $\bar{x}\pm s$ for 10 rats in each group. Differences are shown as follows: ^★^*P* < 0.05 versus control group; ^★★^*P* < 0.01 versus control group; ^▲^*P* < 0.05 versus model group; ^▲▲^*P* < 0.05 versus model group; ^◆^*P* < 0.05 versus acupuncture group; ^◆◆^*P* < 0.01 versus acupuncture group; ^■■^*P* < 0.01 versus fluoxetine group; ^∗^*P* < 0.05 versus Acu + H89.

**Table 3:** Differences showing the effects of stress/antidepressant treatments on the expression levels of hippocampal PKA-*α*, CREB, and p-CREB in depression rats induced by CUMS.

Group	*n*	PKA-*α*/GAPDH	CREB/GAPDH	p-CREB/GAPDH
Control	10	3.277 ± 0.964	1.673 ± 0.226	1.268 ± 0.405
Model	10	2.089 ± 0.614^★★^	1.665 ± 0.107	0.733 ± 0.197^★★^
Acu	10	3.185 ± 0.579^▲▲^	1.666 ± 0.124	1.117 ± 0.211^▲^
FLX	10	3.048 ± 0.425^▲^	1.612 ± 0.366	1.037 ± 0.164
Acu + H89	10	3.057 ± 0.473^▲^	1.478 ± 0.463	0.671 ± 0.295^★★,◆◆^
FLX + H89	10	3.067 ± 0.595^▲^	1.572 ± 0.382	0.619 ± 0.204^★★,■■^

Acu: acupuncture group; FLX: fluoxetine group; Acu + H89: Acupuncture + H89 group; FLX + H89: Fluoxetine + H89 group; PKA-*α*: protein kinase A-*α*; CREB: cyclic adenosine monophosphate response element-binding protein; p-CREB: Phosphor-CREB; GAPDH: glyceraldehyde-3-phosphate dehydrogenase. Differences are shown as follows: ^★★^*P* < 0.01 versus control group; ^▲^*P* < 0.05 versus model group; ^▲▲^*P* < 0.01 versus model group; ^◆◆^*P* < 0.01 versus acupuncture group; ^■■^*P* < 0.01 versus fluoxetine group. Results are presented as $\bar{x}\pm s$ for 10 rats in each group.
